# Is isoprenylcysteine carboxyl methyltransferase the key to reverse ageing?

**DOI:** 10.3389/fnagi.2013.00040

**Published:** 2013-08-14

**Authors:** Danielle Grams, Richard Lockey, Narasaiah Kolliputi

**Affiliations:** Division of Allergy and Immunology, Department of Internal Medicine, Morsani College of Medicine, University of South FloridaTampa, FL, USA

Hutchinson-Gilford progeria (HGPS) is a rare, genetic progeroid disorder that causes premature ageing, nuclear lamina shape abnormalities, growth impairment, and early death at ~13 year of age (Gordon et al., 2013). The disorder is a result of a spontaneous point mutation in the gene lamin A/C (*LMNA)* which encodes for the nuclear lamina scaffold protein, prelamin A. The most common point mutation occurs within exon 11 and results in a silent Gly-to-Gly mutation that causes increased usage of an internal cryptic splice site. This cryptic splice site produces a truncated form of prelamin A known as progerin (Eriksson et al., 2003). In non-mutated cells, prelamin A undergoes a series of modifications to produce lamin A, a vital nuclear lamina structural protein. Prelamin A contains a carboxyterminal *CAAX* motif that is farnesylated on the *CAAX* motif cysteine by farnesyltransferase (FTase). Farnesylation then targets prelamin A to the inner nuclear membrane where the last three amino acids are cleaved by zinc metallopeptidase STE24 (ZMPSTE24). This is followed by immediate methylation of the farnesylcysteine by isoprenylcysteine carboxyl methyltransferase (ICMT) and subsequent cleavage by ZMPSTE24 to produce lamin A. Following cleavage from the nuclear membrane, lamin A is capable of migrating to the nucleoplasm. In HGPS, progerin lacks a vital cleavage site utilized by ZMPSTE24. This results in progerin remaining permanently attached to the inner nuclear membrane and is suspected to contribute the HGPS phenotype (Fantle et al., 1994; Davies et al., 2009).

Although previous studies have been dedicated to treating HGPS by halting farnesylation via FTase inhibitors, Ibrahim et al. targeted *Icmt* expression as a means to reverse progeria-like symptoms in a mouse model of HGPS (Ibrahim et al., 2013). Methylation of other *CAAX* protein motifs has been shown to play a role in protein membrane targeting (Bergo et al., 2002; Michaelson et al., 2005). To explore the role of *CAAX* methylation in HGPS, Ibrahim et al. introduced a hypomorphic allele of *Icmt* into a mouse model of HGPS that utilizes *Zmpste24* deficient mice. It was found that the mice hypomorphic for *Icmt* had increased body weight, normalized grip strength, decreased bone fractures and decreased death compared to non-hypomorphic mice.

Analysis of primary mouse embryonic fibroblasts obtained from sacrificed mice indicate that reduced ICMT activity does not affect levels of prelamin A, but instead causes mislocalization of prelamin A away from the nuclear rim. Interestingly, there is not a subsequent reduction in the number of misshapen nuclei in *Icmt* hypomorphic mice fibroblasts. This indicates that misshapen nuclei play a lesser role in the HGPS phenotype than previously thought. *Icmt* hypomorphic mice fibroblasts also restored cell proliferation to rates similar to wild type fibroblasts. To further elucidate the role of ICMT within cell proliferation, Ibrahim et al. evaluated the impact of ICMT on the on AKT-mTOR cell growth, proliferation and survival pathway. It was found that reduced ICMT activity triggers prelamin A dependent activation of AKT-mTOR, which decreases premature senescence of *Zmpste24* deficient fibroblasts. Furthermore, ICMT activates the pathway through AKT and not mTOR. The precise mechanism of interaction was not determined.

The findings of the Ibrahim et al. study are interesting not only to the field of progeroid disorders, but to the field of ageing studies at large. Scaffidi and Misteli found that dermal fibroblasts with wild type *LMNA* are capable of utilizing the cryptic spice site seen in HGPS cells. The truncated protein was not found to accumulate with age, but the localization of the protein shifted from the nucleoplasm to the nuclear rim with increasing age (Scaffidi and Misteli, 2006). Other studies have found mixed results with some reporting a direct correlation between progerin accumulation and age and others finding no association (McClintock et al., 2007; Cao et al., 2011). Progerin accumulation has also been linked to telomere dysfunction in normal human fibroblasts. Cells utilizing the *LMNA* cryptic splice site have shorter telomeres and high senescence-associated β-gal activity (Cao et al., 2011).

Thus, the *LMNA* splice site plays a critical role not only in HGPS but also in normal ageing and cellular senescence. The Ibrahim et al. study has provided promising results for preventing progerin accumulation at the nuclear rim by reducing ICMT activity. Finding endogenous and exogenous mediators that can control the ICMT activity seen in aging conditions is an important step to discover pharmaceutical intervention and even possible reverse aging. Understanding the influence of ICMT in aging is likely to provide important insights that will not only guide investigation of the molecular and cellular basis of aging, but may also help to identify novel treatment strategies targeting these pathways.
